# Microbial Communities Shaped by Treatment Processes in a Drinking Water Treatment Plant and Their Contribution and Threat to Drinking Water Safety

**DOI:** 10.3389/fmicb.2017.02465

**Published:** 2017-12-12

**Authors:** Qi Li, Shuili Yu, Lei Li, Guicai Liu, Zhengyang Gu, Minmin Liu, Zhiyuan Liu, Yubing Ye, Qing Xia, Liumo Ren

**Affiliations:** State Key Laboratory of Pollution Control and Resource Reuse, College of Environmental Science and Engineering, Tongji University, Shanghai, China

**Keywords:** microbial community, Illumina MiSeq sequencing, function prediction, drinking water treatment processes, aquatic pathogens, chlorine resistant bacterial populations

## Abstract

Bacteria play an important role in water purification in drinking water treatment systems. On one hand, bacteria present in the untreated water may help in its purification through biodegradation of the contaminants. On the other hand, some bacteria may be human pathogens and pose a threat to consumers. The present study investigated bacterial communities using Illumina MiSeq sequencing of 16S rRNA genes and their functions were predicted using PICRUSt in a treatment system, including the biofilms on sand filters and biological activated carbon (BAC) filters, in 4 months. In addition, quantitative analyses of specific bacterial populations were performed by real-time quantitative polymerase chain reaction (qPCR). The bacterial community composition of post-ozonation effluent, BAC effluent and disinfected water varied with sampling time. However, the bacterial community structures at other treatment steps were relatively stable, despite great variations of source water quality, resulting in stable treatment performance. Illumina MiSeq sequencing illustrated that *Proteobacteria* was dominant bacterial phylum. Chlorine disinfection significantly influenced the microbial community structure, while other treatment processes were synergetic. Bacterial communities in water and biofilms were distinct, and distinctions of bacterial communities also existed between different biofilms. By contrast, the functional composition of biofilms on different filters were similar. Some functional genes related to pollutant degradation were found widely distributed throughout the treatment processes. The distributions of *Mycobacterium* spp. and *Legionella* spp. in water and biofilms were revealed by real-time quantitative polymerase chain reaction (qPCR). Most bacteria, including potential pathogens, could be effectively removed by chlorine disinfection. However, some bacteria presented great resistance to chlorine. qPCRs showed that *Mycobacterium* spp. could not be effectively removed by chlorine. These resistant bacteria and, especially potential pathogens should receive more attention. Redundancy analysis (RDA) showed that turbidity, ammonia nitrogen and total organic carbon (TOC) exerted significant effects on community profiles. Overall, this study provides insight into variations of microbial communities in the treatment processes and aids the optimization of drinking water treatment plant design and operation for public health.

## Introduction

Bacteria play an important role in water treatment. On one hand, bacteria from untreated water can utilize organic and inorganic matters as growth substrates, resulting in enhanced biological stability and lower levels of micropollutants in water (Lautenschlager et al., 2013; Hedegaard and Albrechtsen, 2014). On the other hand, some may be potential human pathogens, such as some *Legionella* (Berjeaud et al., 2016) and *Mycobacterium* species (Vaerewijck et al., 2005). Making full use of bacterial biodegradation and controlling pathogens are thus two major goals in drinking water treatment.

Biofiltration, one of the oldest water treatment methods, is designed to encourage bacterial growth on granular materials to enable biodegradation (Proctor and Hammes, 2015). Biofiltration processes (e.g., rapid sand, granular activated carbon (GAC), and slow sand filtration) are categorized according to their support materials and operation modes. Sand and GAC filtration are the most popular methods, and are regarded as conventional and advanced treatments, respectively. Biofiltration performance depends on stable bacterial community structures and high microbial activity (Fonseca et al., 2001; Kim et al., 2014), but treatment processes and their configurations usually cause variations in the bacterial community (Zeng et al., 2013; Prest et al., 2016). The bacterial communities present in treatment systems were mainly introduced by the source water (Yang et al., 2011). In general, the first two treatments applied to source water are flocculation and sedimentation that do not significantly change the microbial community structure (Lin et al., 2014). This is followed by sand filtration. The bacterial community found in the sand filters is modulated by the bacteria present in the sedimentation effluent (Xu et al., 2017). GAC filtration usually comes next, most of the times combined with ozone which constitutes an ozone-biological activated carbon (O_3_-BAC) treatment process. Ozone oxidizes natural organic matters, forming easily biodegradable, low-molecular-weight by-products, and increases dissolved oxygen (DO) concentrations. Ozone is thus positively correlated with the growth of microorganisms on GAC (Yang et al., 2016). However, ozone, as a powerful oxidant, can also effectively inactivate bacteria (Hunt and Marinas, 1999). The effect of ozonation on bacterial communities therefore deserves further research, which will facilitate evaluating the influence of ozonation on BAC filtration.

Biofiltration effectively removes biodegradable compounds. However, biofilms colonized on filter materials can slough off, shaping the subsequent bacterial community (Pinto et al., 2012; Lautenschlager et al., 2014) and increasing bacterial populations in the effluent (Stewart et al., 1990; Zhang et al., 2015). Bacteria released from BAC filters are extremely resistant to disinfection (Camper et al., 1986; Yu et al., 2014). Disinfection, the final step of water treatment, is critical to control the microbiome released into the treated water and inhibit microbial growth during distribution. However, disinfection cannot completely destroy the microbiome in treatment plants and thus potentially acts as a stress-based selective pressure (Gomez-Alvarez et al., 2012; Holinger et al., 2014; Wang et al., 2014). The bacteria selected by disinfectants should receive more attention.

Several physicochemical water parameters, as pH, temperature, dissolved organic carbon, etc., were described to influence the bacterial community dynamics (Lindstrom, 2000; Li et al., 2010; Pinto et al., 2012; Kim et al., 2014; Staley et al., 2015). Understanding the correlation between water quality parameters and bacterial communities could help trace changes in microbiome by monitoring water quality parameters. Seasonal changes cause great variations in water quality indices (Li et al., 2014; Feng et al., 2016). However, only a few studies investigated the bacterial community structure in a water treatment plant across the four seasons (Pinto et al., 2012). To study the microbiome structure and diversity, culture-independent methods allow deeper analysis than the culture-dependent ones. The advantage of its long reads once led 454 pyrosequencing to be widely used on drinking water samples (Pinto et al., 2012; Zeng et al., 2013; Kim et al., 2014; Lautenschlager et al., 2014; Lin et al., 2014). However, 454 pyrosequencing has a higher per-base error rate and is susceptible to indel errors in homopolymer stretches (Loman et al., 2012). Therefore, 454 pyrosequencing has been discontinued with the development of next-generation sequencing (NGS) (Tan et al., 2015). Illumina MiSeq technology has become increasingly popular due to its advantages of lower cost, greater throughput and higher accuracy (Hirai et al., 2017). However, applications of Illumina MiSeq sequencing to drinking water remain scarce (LaPara et al., 2015). As different sequencing platforms are generally discrepant (Smith and Peay, 2014; Sinclair et al., 2015), the adoption of new sequencing platforms for drinking water samples is of paramount significance. In addition, phylogenetic investigation of communities by reconstruction of unobserved states (PICRUSt), a new computational approach, enables predicting the functional composition of bacterial communities using 16S rRNA marker gene sequences (Langille et al., 2013). In contrast to the qualitative analysis of high-throughput sequencing, the real-time quantitative polymerase chain reaction (qPCR) is a useful tool for the quantitative tracking of specific bacterial strains (Brinkman et al., 2003). The integration of high-throughput sequencing with qPCR is thus beneficial for assessing the composition of microbial communities, especially for detecting specific bacteria.

The primary objective of this study was to analyze the variations in bacterial communities and functional profiles during treatment processes to determine the role bacteria play in water treatment. Illumina MiSeq sequencing was applied to identify and characterize bacterial communities in a drinking water treatment plant that features conventional and advanced biofiltration processes. To clarify seasonal influence in the bacterial community structure, sampling was carried out at each process step over one year. The obtained 16S data were processed using PICRUSt 1.0.0 to predict the functions of microbial communities. Bacteria from the genera *Mycobacterium* and *Legionella*, which include human pathogens, were quantified using qPCR. In addition, the water quality parameters were measured over the sampling year to access to their influence on the bacterial communities. The proper adjustment of the water quality parameters would allow a better control and management of the bacterial community structures over the drinking water treatment systems.

## Materials and methods

### Drinking water treatment processes

**Figure 1:**
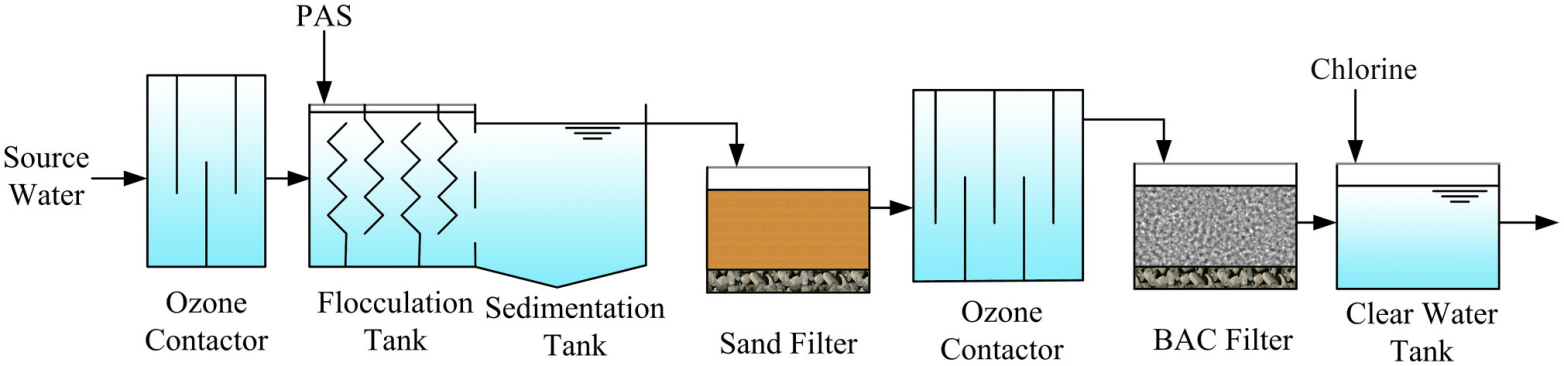
Schematic diagram of the drinking water treatment plant (PAS, polyaluminium sulfate).

The drinking water treatment plant is located in Wujiang District, Suzhou, Jiangsu Province, China (31.11°N, 120.62°E). The source water originated from the eastern Lake Taihu, the third largest freshwater lake in China. Lake Taihu has been experiencing eutrophication problems for several decades. The average water quality of Lake Taihu is ranked as class IV (class I-V, the best to the worst), according to the Environmental Quality Standard for Surface Water of China. The samples were taken from the eastern part of Lake Taihu, where the water quality is the best in the lake. This plant produces nearly 300,000 m^3^/day drinking water. The source water was successively treated by preozonation, flocculation, sedimentation, sand filtration, post-ozonation, BAC filtration and chlorine disinfection (Figure 1). Appropriate amounts of free chlorine were added to the BAC effluent to secure free residual chlorine levels above 0.4 mg/L after 30 min of contact time. At the preozonation step, 0.5 mg/L ozone is added with a contact time of 5 min, while 1 mg/L ozone is added with a 12 min contact time for post-ozonation.

### Sampling, sample processing and physico-chemical analysis

The sampling campaign was conducted during 4 months (November, January, May and July) from 2015 to 2016. Each of the 4 months belonged to a different season (fall, winter, spring and summer, respectively). Water samples were taken in triplicate from each water treatment step in 1 L sterile bottles. BAC and sand media were collected from the upper and middle parts of filter beds in 5 mL sterile sample tubes. Three sampling spots were randomly selected each time that the filters were sampled. The upper biofilm samples were collected from the surface of filters. The middle biofilm samples were collected at a depth of 0.5–0.8 m. All the samples were transported on ice to the laboratory and processed within 12 h. Each water sample was carefully pooled with its three replicates. Then, 0.5 L of surface water, 2 L of disinfected water and 1 L of each for the remaining water samples were filtered through 0.22 μm nitrocellulose membranes (50 mm diameter, Millipore, USA). Three replicate biofilm samples from each part of filters were carefully pooled. The filter membranes and mixed biofilm media were stored at −80°C before DNA extraction.

Temperature, pH and DO were measured immediately after water sampling at each sampling site using an HQ30d portable multi-parameters water quality analyzer (Hach, USA). Ammonia nitrogen, nitrite and nitrate were analyzed according to standard protocols (APHA, 2011). Turbidity was determined using a 2100N turbidity analyzer (HACH, USA). Total organic carbon (TOC) was measured using an Aurora 1030W TOC analyzer (OI, USA).

### DNA extraction, PCR amplification and sequencing

The filtered membranes with collected biomass were cut into pieces with sterilized scissors, and total DNA was extracted using an E.Z.N.ATM Mag-Bind Soil DNA Kit (OMEGA, USA) following the manufacturer's protocol. Appropriate biofilm samples (0.5 g of BAC samples and 2 g of sand samples) were subjected to DNA extraction using this kit. The bacterial V3-V4 region of the 16S rRNA gene was amplified using the forward primer 341F (5′-CCCTACACGACGCTCTTCCGATCTG−3′) and the reverse primer 805R (5′-GACTGGAGTTCCTTGGCACCCGAGAATTCCA-3′). PCR amplification was performed by a T100™ Thermal Cycler (BIO-RAD, USA). All PCR reactions were performed in triplicate with 20 μL of the final reaction mixture, which contained 4 μL of 5 × Fast Pfu Buffer, 2 μL of 2.5 mM dNTPs, 0.8 μL of each primer (5 μM), 0.4 μL of Taq DNA Polymerase (Thermo Scientific, USA), 0.2 μL BSA, and 10 ng of template DNA. The thermal cycling conditions were as follows: an initial denaturation step at 95°C for 3 min, and 28 cycles at 95°C for 30 s, 55°C for 30 s, and 72°C for 45 s, followed by a final extension at 72°C for 10 min (Shu et al., 2016). PCR products were initially screened using 2% agarose gels and purified using Agencourt AMPure XP (Beckman, USA) according to the instructions. Purified PCR products were paired-end sequenced on the Illumina MiSeq platform (Illumina, USA) at a read length of 2 × 300 bp. In total, 44 samples were sequenced at the Sangon Biotech (Shanghai) Co. Ltd.

The 16S rRNA gene sequences were deposited in the NCBI Sequence Read Archive under accession number SRP106506.

### Real-time quantitative PCR (qPCR) analysis for potential pathogens and total bacteria

*Legionella* spp. *Mycobacterium* spp. and total bacteria were enumerated by qPCR using an ABI7500 Real-Time PCR System (Life Technologies, USA). For *Legionella* spp., a PCR strategy was designed to amplify the *Legionella* genus-specific 23S rRNA gene, using the forward primer 5′-CCCATGAAGCCCGTTGAA-3′ and the reverse primer 5′-ACAATCAGCCAATTAGTACGAGTTAGC-3′, with the 5′-HEX-TCCACACCTCGCCTATCAACGTCGTAGT-TAMRA-3′ TaqMan probe (Nazarian et al., 2008). The PCR program was as follows: 95°C for 30 s, and 40 cycles at 95°C for 5 s and 58.5°C for 34 s.

*Mycobacterium* spp. were quantified by targeting 16S rRNA gene using the forward primer (5′-CCTGGGAAACTGGGTCTAAT-3′), the reverse primer (5′-CGCACGCTCACAGTTA-3′) and probe (5′-FAM-TTTCACGAACAACGCGACAAACT-TAMRA-3′) (Radomski et al., 2010). The PCR program was as follows: 95°C for 30 s, and 45 cycles at 95°C for 5 s, 55°C for 15 s and 72°C for 34 s. Total bacteria were quantified using the forward primer (1369F: CGGTGAATACGTTCYCGG) and the reverse primer (1492R: GGWTACCTTGTTACGACTT) with an annealing temperature of 55°C (Suzuki et al., 2000).

qPCR reactions were performed in triplicate with each 10-μl reaction mixture containing 5 μl of 2 × Premix Ex Taq, 200 nM each primer, 100 nM probe, 0.1 μl of 50 × ROX as a reference dye and 1 μl of DNA template. Control reactions contained the same mixtures, but with 1 μl of sterile water replacing the DNA template. The standard DNA were prepared and run during each qPCR to generate standard curves (*r*^2^ > 0.99). Amplification efficiency was monitored and standards amplified with similar efficiencies (91–99%, depending on the assay).

### Sequence processing and data analysis

The sequence data were processed to trim the reads with a Q_phred_ score below 20 using QIIME (Caporaso et al., 2010). Adapters were removed using cutadapt (Chen et al., 2014). Trimmed paired-end reads were merged with a maximum mismatch rate of 1 mismatch in 10 bases using PEAR (Unno, 2015). Then sequences were demultiplexed using QIIME. And the quality filtering was performed using Prinseq (Schmieder and Edwards, 2011) to remove homopolymers longer than 8 bp and sequences less than 200 bp, showing ambiguous base “N” or with average base quality score less than 20. UCHIME software was used to identify and remove chimeras (Edgar et al., 2011). All samples were normalized to ensure an equal number of sequences in each sample by random subsampling for further analyses. Operational Taxonomic Units (OTUs) were clustered with a 97% similarity cutoff using Usearch (Edgar, 2010) and classified against the Ribosomal Database Project (RDP) dataset with a confidence level cutoff of 80% (Cole et al., 2009). Sequences flagged as chloroplasts, mitochondria, or eukaryotes (accounting for 1.8% of all sequences) were excluded. Mothur ver. 1.30.1 (Schloss et al., 2009) was used to calculate bacterial diversity indices (Shannon, Simpson, abundance-based coverage estimator (ACE), Chao1, and coverage). Heatmap was implemented by R packages heatmap (http://www.r-projet.org/) (Lin et al., 2014). R vegan package was used to estimate the weighted UniFrac metric and to perform principal coordinate analysis (PCoA) (Noyce et al., 2016). Redundancy analysis (RDA) was employed to explore the relationship between environmental factors and bacterial communities. One way analysis of variance (ANOVA) tests were performed to assess the statistically significant difference of diversity indices between samples. Differences were considered significant at *p* < 0.05. Venn diagrams were drawn using online tool “Draw Venn Diagram” (http://bioinformatics.psb.ugent.be/webtools/Venn) to analyze overlapped and unique OTUs during the treatment processes. One-way permutational analysis of variance (PERMANOVA) was performed using R vegan package to assess the statistically significant effects of treatment processes on bacterial communities (Anderson and Walsh, 2013).

### Prediction of functional profiles

For functional composition prediction, PICRUSt-compatible OTU tables were constructed using the closed-reference OTU-picking strategy (pick_closed_ referfence _otus. py, uclust similarity cutoff at 0.97, Greengene v13_8) in QIIME 1.8.0 (Langille et al., 2013). The resulting OTU table was uploaded into the PICRUSt 1.0.0 on the Galaxy server (http://huttenhower.sph.harvard.edu/galaxy/). The functional information were annotated according to the Kyoto Encyclopedia of Genes and Genomes (KEGG) database. The values of Nearest Sequenced Taxon Index (NSTI) for all the samples were between 0.10 and 0.24, indicating relatively accurate predictions. The non-metric multidimensional scaling (NMDS) plot was made using R vegan package based on PICRUSt analysis.

## Results and discussion

### Physicochemical properties of water

**Figure 2:**
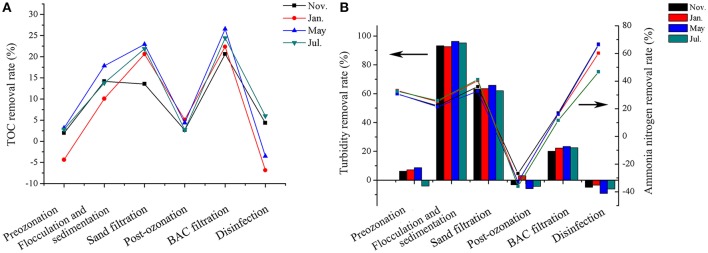
Variations in removal rates of TOC **(A)**, turbidity (left axes, histogram) and ammonia nitrogen (right axes, line graph) **(B)** along the treatment processes at different times. The arrows in the figure indicated the corresponding vertical coordinate of different data.

The water quality parameters of the source and treated water at each sampling time are shown in Table 1. All the water quality parameters varied, but the treated water always met the water quality standard (GB5749-2006). The TOC removal rates during the treatment processes are shown in Figure 2A. Flocculation-sedimentation, sand filtration and BAC filtration were important processes for TOC removal and their TOC removal rates relatively stabilized at 10–15%, 20% and more than 20%, respectively. The rates of turbidity and ammonia nitrogen removal in each treatment process were illustrated in Figure 2B. More than 90% of turbidity was removed by flocculation-sedimentation. The turbidity of all samples was below 1 NTU after flocculation-sedimentation. Sand filtration and BAC filtration subsequently removed approximately 60 and 20% of turbidity, respectively. All the treatment processes except for post-ozonation contributed to the removal of ammonia nitrogen. Post-ozonation increased the concentration of ammonia nitrogen by approximately 30%, which may be due to the products of nitrogenous organic matter decomposition (LeLacheur and Glaze, 1996). Chlorine disinfection had the highest rate (47–66%) of ammonia nitrogen removal because of the reaction of chlorine and ammonia nitrogen (Hayes-Larson and Mitch, 2010). The removal rates of all the parameters did not significantly differ among different seasonal samples (*p* > 0.05).

**Table 1:** Water quality parameters of source water and treated water at each sampling time.

**Sample by type and date**	**Temperature (°C)**	**pH**	**Turbidity (NTU)**	**DO (mg/L)**	**TOC (mg/L)**	**Nitrate (mg/L, as ${\text{NO}}_{3}^{-}$)**	**Nitrite (mg/L, as ${\text{NO}}_{2}^{-}$)**	**Ammonia (mg/L, as ${\text{NH}}_{3}^{-}$N)**
**RAW WATER**								
Nov.	16.7	7.83	10.7	9.30	4.97	0.76	0.038	0.46
Jan.	6.2	8.13	12.5	12.36	4.26	0.82	0.035	0.48
May	20.3	7.76	26.1	7.7	8.26	0.72	0.043	0.44
Jul.	28.6	7.68	19.4	6.32	8.55	1.53	0.042	0.40
**TREATED WATER**								
Nov.	16.5	7.51	0.23	10.16	2.61	0.81	No detected^a^	0.06
Jan.	6.8	7.46	0.30	12.34	2.50	0.78	0.003	0.05
May	20.1	7.43	0.25	9.03	3.68	0.91	No detected^a^	0.03
Jul.	28.3	7.25	0.28	7.26	3.38	1.68	0.002	0.07

a *No Detected: below the detection limit*.

### Effects of treatment processes on bacterial communities

The community richness and diversity indices at each treatment step during seasonal sampling are shown in Table 2. With a 97% similarity cutoff, 22,522, 10,142, 12,345, and 17,132 OTUs were acquired in November, January, May and July, respectively. After post-ozonation, the Chao1 and Shannon indices respectively increased from 1,335–3,133 to 1,428–4,258 and from 3.74–4.59 to 4.54–6.37 (*p* > 0.05). Chlorine disinfection reduced the richness and diversity indices (Chao1 and Shannon indices) from 2,469–5231 to 1,300–4,237 and from 5.62–6.91 to 4.15–6.13 (*p* > 0.05), respectively. The influence of treatment processes on the richness and diversity of bacterial community was not significant. The richness and diversity of the bacterial community varied in each process step with sampling time. However, associations between these variables were not found.

**Table 2:** Bacterial community richness and diversity indices at each treatment step and the four sampling times.

**Sample by type and date**		**OTUs**	**Coverage**	**ACE**	**Chao1**	**Shannon**	**Simpson**
RW	Nov.	1486	0.944	5800	3451	4.90	0.032
	Jan.	834	0.976	2517	1659	4.58	0.024
	May	1480	0.963	3292	2502	5.04	0.027
	Jul.	1147	0.971	2830	2078	4.53	0.042
PROE	Nov.	1508	0.952	4697	3208	4.49	0.044
	Jan.	840	0.976	2347	1674	4.55	0.026
	May	1448	0.961	3594	2652	5.05	0.024
	Jul.	1522	0.959	3802	2836	4.94	0.030
SE	Nov.	2459	0.926	6093	4289	5.28	0.031
	Jan.	586	0.983	1796	1264	3.98	0.041
	May	608	0.983	1717	1285	3.62	0.074
	Jul.	957	0.973	2950	1973	3.23	0.193
SFE	Nov.	1643	0.951	4730	3133	4.59	0.038
	Jan.	649	0.983	1782	1335	4.20	0.034
	May	856	0.978	2134	1590	3.74	0.074
	Jul.	1477	0.961	3732	2662	3.78	0.168
PSOE	Nov.	2878	0.925	4738	4258	6.37	0.008
	Jan.	766	0.981	1760	1428	4.58	0.030
	May	1277	0.970	2558	2093	4.54	0.049
	Jul.	2630	0.940	4146	3884	5.89	0.021
BACE	Nov.	3402	0.910	6807	5231	6.91	0.003
	Jan.	1527	0.964	3184	2469	5.62	0.010
	May	1883	0.959	3677	2944	5.86	0.012
	Jul.	2181	0.953	4088	3326	6.15	0.010
DW	Nov.	3011	0.917	4733	4237	6.13	0.019
	Jan.	964	0.979	1425	1300	4.15	0.053
	May	1609	0.963	2558	2297	4.77	0.036
	Jul.	2612	0.946	3751	3444	5.78	0.036
USB	Nov.	1185	0.967	3406	2245	4.46	0.038
	Jan.	939	0.976	2349	1741	4.49	0.035
	May	655	0.986	1413	1047	4.10	0.045
	Jul.	1065	0.977	2256	1804	5.01	0.023
MSB	Nov.	990	0.974	2644	1914	4.36	0.048
	Jan.	806	0.981	1826	1437	4.45	0.034
	May	641	0.985	1574	1057	4.09	0.041
	Jul.	954	0.978	2357	1787	4.76	0.032
UBACB	Nov.	2167	0.946	4675	3375	6.04	0.011
	Jan.	1033	0.978	1982	1724	4.59	0.065
	May	974	0.981	1706	1427	4.82	0.037
	Jul.	1144	0.975	2321	1837	4.88	0.025
MBACB	Nov.	1793	0.956	4127	3011	5.75	0.014
	Jan.	1198	0.977	2075	1693	5.00	0.049
	May	914	0.981	1697	1520	4.44	0.064
	Jul.	1443	0.970	2779	2233	5.57	0.013

*RW, raw water; PROE, preozonation effluent; SE, sedimentation effluent; SFE, sand filtration effluent; PSOE, post-ozonation effluent; BACE, biological activated carbon effluent; DW, disinfected water; USB, upper sand biofilm; MSB, middle sand biofilm; UBACB, upper biological activated carbon biofilm; MBACB, middle biological activated carbon biofilm*.

The effects of the major treatment process steps on the bacterial communities were investigated using Venn diagrams (Figure S1). In total, 54–193 OTUs were universally present from raw water to disinfected water at different sampling times. The percentages of the total number of OTUs that are common during the treatment process were 1.5, 1.1, 1.0, and 1.1% in November, January, May and July, respectively, indicating that the common OTUs accounted for a very small portion of the OTUs detected. Samples from each major-step, namely sand filtration, post-ozonation, BAC filtration and chlorine disinfection, contained 22–67% unique OTUs (Figure S1). The percentage of unique OTUs was largest in the effluent of the BAC filter and disinfected water (40–53% and 36–67%, respectively), indicating that BAC filtration and disinfection had power to shape the microbiome. The effluent from sand filtration had the lowest proportion of unique OTUs (22–27%), which implied that sand filtration exerted a weaker influence on the bacterial community than the other major process steps.

PERMANOVA revealed that no significant differences existed between the bacterial community structures in two successive sampling sites, namely raw water and preozonation effluent (*F* = 0.32, *P* = 0.836), preozonation and sedimentation effluents (*F* = 2.03, *P* = 0.069), sedimentation and sand filtration effluents (*F* = 0.44, *P* = 0.791), sand filtration and post-ozonation effluents (*F* = 1.16, *P* = 0.296), and post-ozonation and BAC filtration effluents (*F* = 1.26, *P* = 0.163). Similarly, the differences of bacterial community structures between preozonation and sand filtration and between sedimentation and post-ozonation were not significant (*P* > 0.05). Except for the mentioned above, the differences between the bacterial community structures of random two samples were significant (*P* < 0.05). For sequential water samples, only BAC effluent and disinfected water exhibited a significant difference (*F* = 1.73, *P* = 0.027), indicating that all the individual treatment processes except disinfection had no significant effects on the bacterial community. Conversely, the bacterial community was shaped by a combination of multistep processes.

### Variations in bacterial community composition during treatment processes

**Figure 3:**
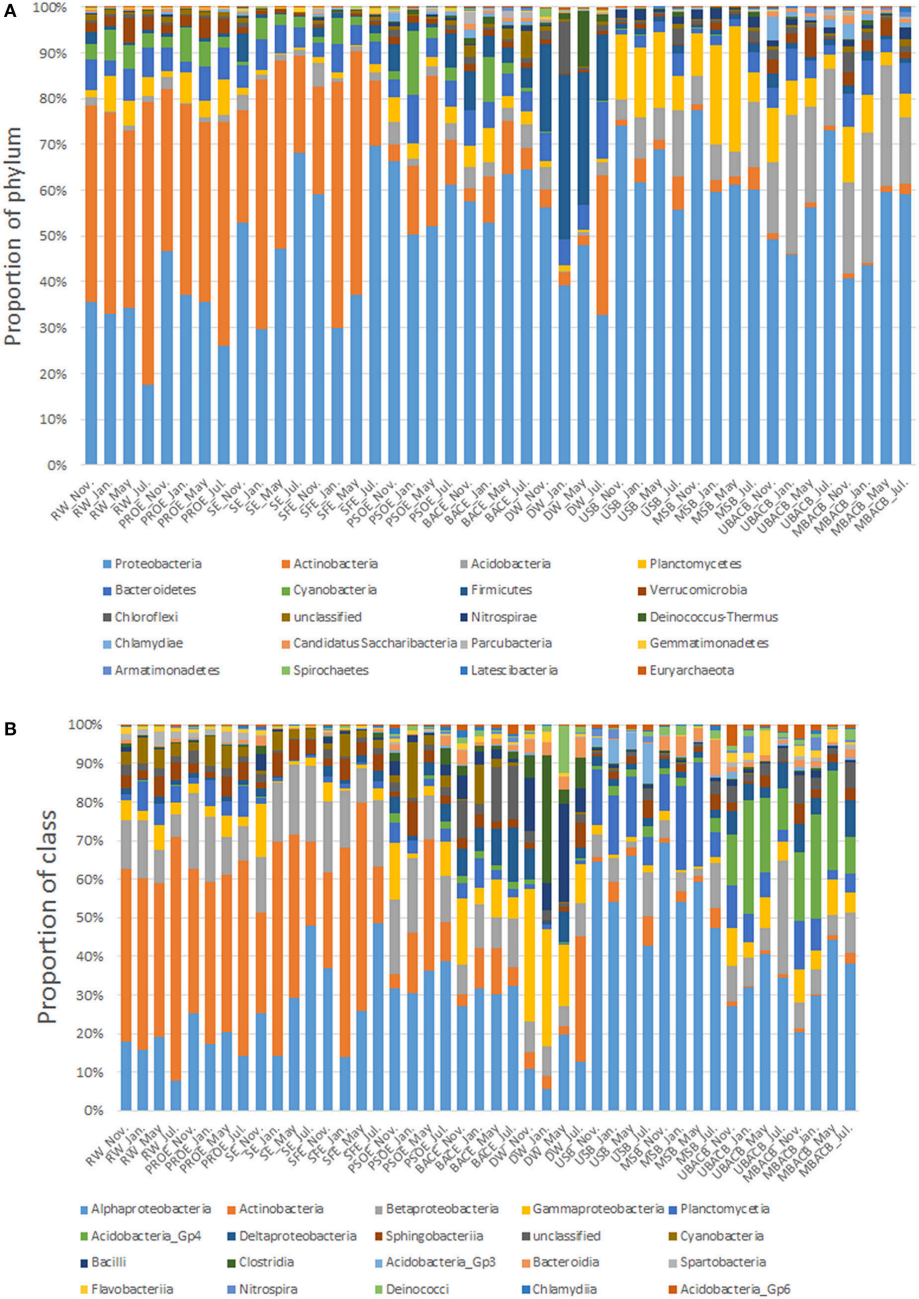
Relative abundance of bacterial phyla **(A)** and classes **(B)** at each treatment step. The top 20 most abundant phyla or classes are shown. The abbreviations are the same as used in Table 2.

The top 20 most abundant phyla of bacterial community detected at the 11 sampling locations and four sampling times is shown in Figure 3A. The bacterial community composition in raw water, preozonation effluent, sedimentation effluent, sand filtration effluent and biofilm samples remained relatively stable despite the variations in proportions of *Proteobacteria* and *Actinobacteria* during seasonal sampling. However, the bacterial community composition in post-ozonation, BAC filtration effluent and disinfected water samples varied at different sampling times. The predominant phyla, other than *Proteobacteria* (38.54–64.96%), in post-ozonation samples differed in different seasonal samples. They were *Firmicutes* (5.73%), *Planctomycetes* (5.29%) and *Acidobacteria* (4.7%) in November, *Proteobacteria* (38.54%), *Actinobacteria* (11.46%) and *Cyanobacteria* (34.14%) in January, *Actinobacteria* (32.26%) in May and *Actinobacteria* (9.64%), *Firmicutes* (7.44%) and *Bacteroidetes* (5.73%) in July. The predominant bacteria in BAC filtration effluent similarly varied and were *Firmicutes* (8.38%) and *Bacteroidetes* (7.66%) in November; *Cyanobacteria* (18.65%) and *Actinobacteria* (9.08%) in January; *Actinobacteria* (11.47%) and *Bacteroidetes* (4.87%) in May and *Acidobacteria* (5.13%) and *Actinobacteria* (4.64%) in July, in addition to the most predominant phylum overall (*Proteobacteria*, 47.64–64.01%). Compared with the groups in post-ozonation and BAC filtration effluents, the predominant bacterial groups in disinfected water were relatively consistent (*Proteobacteria* at 32.19–55.3%, *Firmicutes* at 14.07–34.56%), except for a high level of *Deinococcus-Thermus* (11.71%) in May and *Actinobacteria* (30.14%) in July.

*Proteobacteria, Actinobacteria*, and *Firmicutes* dominated in different samples. *Actinobacteria* and *Proteobacteria* dominated in raw water, constituting 37.7–61.4% and 17.3–34.9% of the total sequences, respectively. The proportion of *Actinobacteria* gradually decreased along the sequential steps of preozonation, flocculation, sedimentation and sand filtration, while the proportion of *Proteobacteria* correspondingly increased. The abundance of *Actinobacteria* dramatically decreased from 14.3–52.7% to 3.4–32.3% after post-ozonation, resulting in the absolute predominance of *Proteobacteria*. Unlike *Actinobacteria, Proteobacteria* dominated in all the samples, including those of water and biofilms. Chlorine disinfection decreased the proportion of *Proteobacteria* from 47.6–64.0% to 32.19–55.3%. In contrast, the proportion of *Firmicutes* increased from 3.4–8.4% to 14.1–34.6% after disinfection.

The top 20 most abundant classes of bacterial community at all the sampling locations is presented in Figure 3B. Similar to the variations in phyla, the class-level bacterial composition of post-ozonation, BAC filtration effluent and disinfected water differed with seasons. *Proteobacteria* is comprised of five subclasses, *Alpha-, Beta-, Gamma-, Delta-*, and *Epsilonproteobacteria*. *Alphaproteobacteria* was the most prevailing subclass within *Proteobacteria* in all the samples (accounting for approximately 50% of *Proteobacteria*), followed by *Betaproteobacteria* (approximately 30%), except in disinfected water. In disinfected water, the proportion of *Gammaproteobacteria* (~30%) within *Proteobacteria* approached that of *Alphaproteobacteria* (~35%) in May and July and was six to seven times than that of *Alphaproteobacteria* (~15%) in November and January. In addition to *Gammaproteobacteria, Bacilli* and *Clostridia* dominated in disinfected water. They both belong to *Firmicutes* and accounted for similar proportions (5.75–24.15% and 5.32–28.18%, respectively) of the bacterial classes in disinfected water.

*Proteobacteria*, the dominant phylum in all the samples, is the most common bacteria in freshwater lakes. Of the five subclasses of *Proteobacteria, Alphaproteobacteria* predominated because of its competitive advantages under conditions of low nutrient availability and its capability to degrade complex organic compounds (Eiler et al., 2003; Hutalle-Schmelzer et al., 2010). By contrast, *Betaproteobacteria* prefer to proliferate in nutrient-rich environments (Newton et al., 2011), which leads to its weak competitiveness in oligotrophic environments. Although *Gammaproteobacteria* was only abundant in disinfected water, it should receive more attention because of its increase after chlorine disinfection. The dominance of *Gammaproteobacteria* in disinfected water was possibly due to its great resistance to chlorine (Mi et al., 2015; Belila et al., 2016; Pang et al., 2016; Stanish et al., 2016).

*Actinobacteria* are commonly found and often numerically important in a variety of freshwater habitats (Glöckner et al., 2000; Zwart et al., 2002). *Actinobacteria* accounted for the largest proportion of bacterial phyla in raw water. However, they decreased during the treatment processes, implying their vulnerability to treatment (Servais et al., 1994). In contrast, *Bacteroidetes* maintained at a constant level in all the samples, reflecting its resistance to treatment processes. The proportion of *Firmicutes* increased after chlorine disinfection, which may be attributed to the greater resistance of Gram-positive bacteria (*Firmicutes*) than Gram-negative bacteria (*Proteobacteria*) to chlorine (Mir et al., 1997).

### Characteristics of bacterial communities from water and different filters

**Figure 4:**
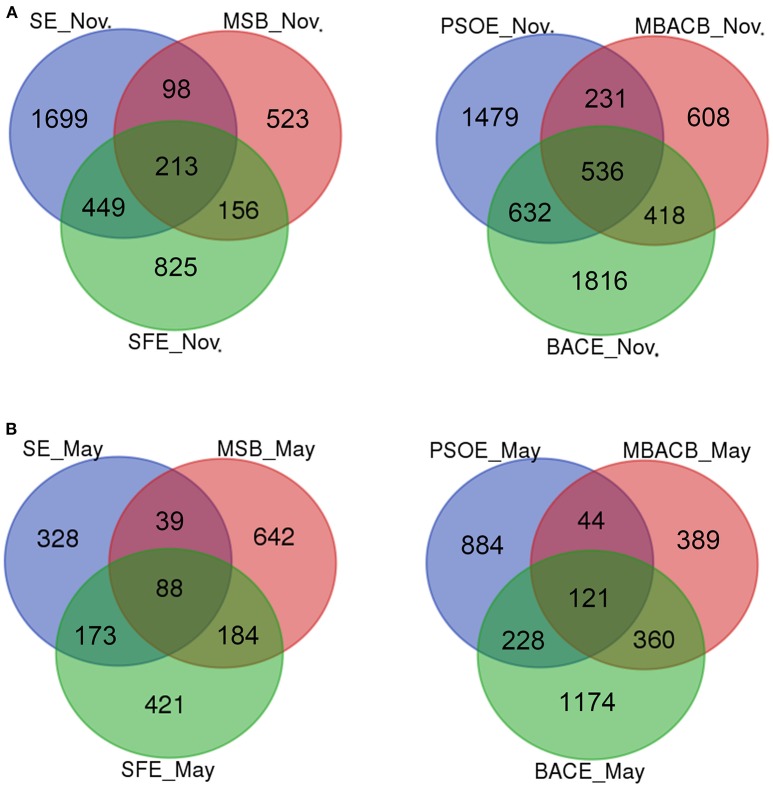
Venn diagrams showing the number of shared OTUs between filter biofilms and their corresponding influents and effluents in November **(A)** and May **(B)**. The abbreviations are the same as used in Table 2.

Species richness indices did not differ in water and biofilm samples (Table 2). The bacterial diversity estimators of BAC biofilms were higher than that of sand biofilms during the sampling time, with a larger number of OTUs (914–2167) and higher values of Chao1 (1427–3375) and Shannon indices (4.44–6.04) in BAC biofilms than sand biofilms [641–1185 OTUs, Chao1 indices (1047–2245) and Shannon indices (4.09–5.01)] (Table 2). However, the differences were not significant (*p* > 0.05). Both sand and BAC biofilms shared more OTUs with their corresponding effluents (28.6–56.3% and 25.6–59.4% of biofilm samples, respectively) than with their respective influents (13.4–32% and 16.8–47.3% of biofilm samples, respectively) (data of November and May shown in Figure 4, and data of January and July shown in Figure S2), which implied that filter media selected for surface-associated bacteria and affected the subsequent flow, despite being seeded by the influent (Dang and Lovell, 2016).

Bacterial community structures of biofilm samples were significantly different from those of water samples (PERMANOVA, *P* < 0.05). The bacterial community composition was similar between the upper and middle parts of each type of filter (sand filter, PERMANOVA, *F* = 0.20, *P* = 0.823; BAC filter, PERMANOVA, *F* = 0.37, *P* = 0.877). However, there was a significant difference in bacterial communities between sand and BAC biofilms (upper part, PERMANOVA, *F* = 5.37, *P* = 0.029; middle part, PERMANOVA, *F* = 5.30, *P* = 0.024). The *Actinobacteria* population, which were dominant in water, became the minor group in biofilms (less than 8%) (Figure 3A). Similarly, the abundance of *Cyanobacteria* was higher in water (1–15%) than biofilms (<0.1–2%). Their low abundance on filter materials indicated that they did not tend to form biofilms (Parfenova et al., 2013). In contrast, *Acidobacteria* and *Planctomycetes*, which were present in low proportions in water (no more than 7% each), were relatively abundant in biofilms. *Acidobacteria* accounted for 4.4–14.4% and 12.2–30.0% of the phyla in sand and BAC biofilms, respectively, while the proportions of *Planctomycetes* were 6.4–27.3% and 2.8–12.1%. Within *Acidobacteria, Acidobacteria_Gp4* (59.8–95.8% of *Acidobacteria*) predominated in BAC biofilms, while *Acidobacteria_Gp3* (54.2–69.8% of *Acidobacteria*) dominated in sand biofilms (Figure 3B). The predominance of *Acidobacteria* and *Planctomycetes* in biofilms may be due to their physiological and genetic traits related to surface living (Dang and Lovell, 2016), and they are widely distributed in drinking water biofilms (Schmeisser et al., 2003; Martiny et al., 2005; Qin et al., 2007). In contrast, the abundance of *Bacteroidetes* differed little between planktonic and sessile forms, accounting for 2.3–7.3%.

### Quantitative analysis of *Mycobacterium* spp. and *Legionella* spp.

**Figure 5:**
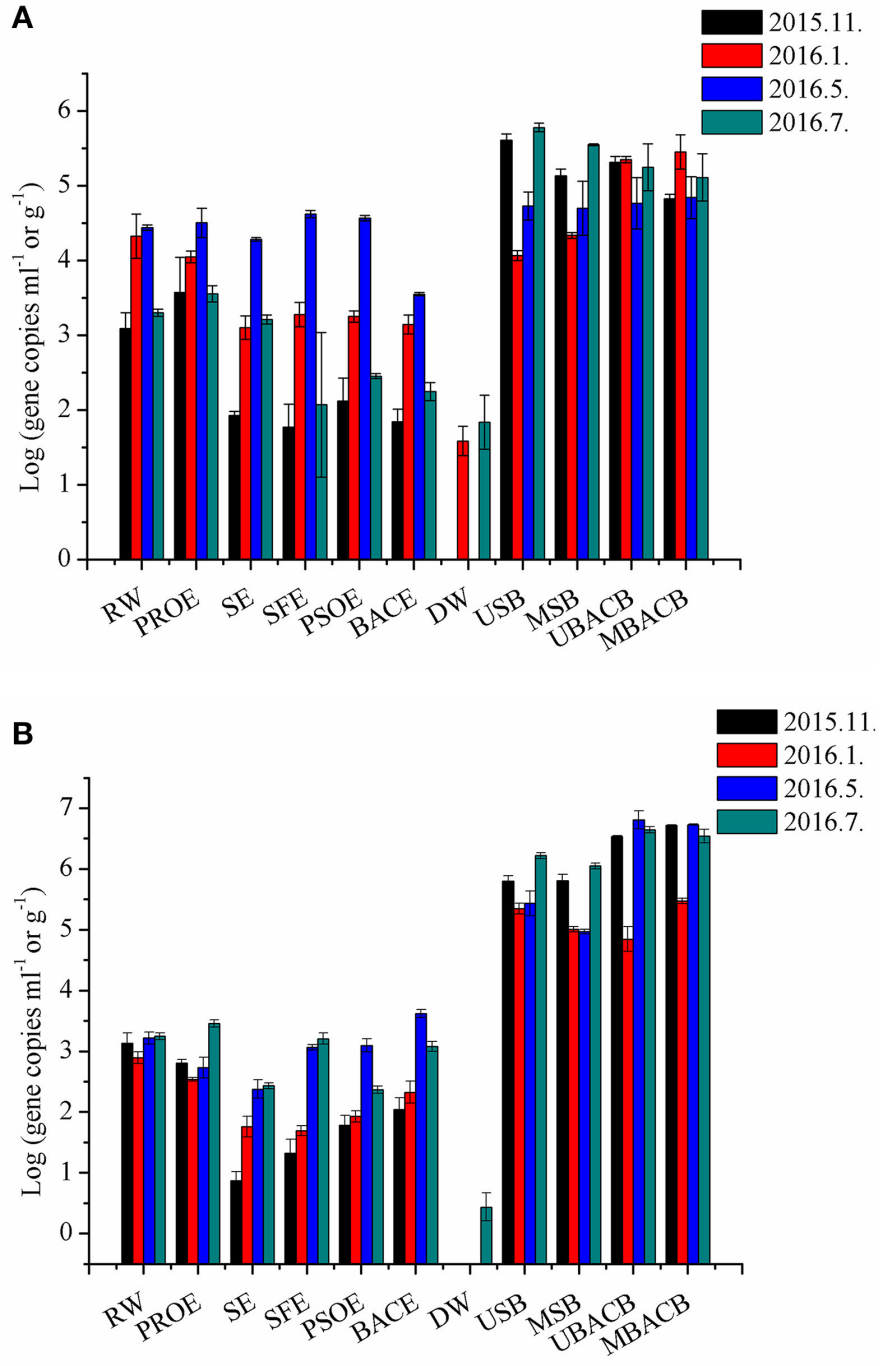
QPCR results for *Mycobacterium* spp. **(A)** and *Legionella* spp. **(B)** at each treatment step during seasonal sampling. The abbreviations of the samples are the same as used in Table 2.

The genus *Mycobacterium*, well known for its resistance to disinfectants (Simoes and Simoes, 2013), was present in a higher proportion (7.24%) in post-ozonation in May and disinfected water (8.30%) in January (Figure S3A). However, the proportion that *Mycobacterium* spp. accounted for in disinfected water varied with seasonal changes significantly (*p* < 0.05). Both *Mycobacterium* spp. and *Legionella* spp. had more gene copies in biofilms than water (Figure 5). However, *Mycobacterium* spp. accounted for quite a small proportion in biofilms, compared to water (Figure S3A). In contrast, *Legionella* spp. presented the accumulation on BAC filters (Figure S3B), resulting in their increase in BAC effluents, which posed a potential threat to consumers. Fortunately, chlorine disinfection effectively eliminated *Legionella* spp. (*p* < 0.05).

Ozone is reported to be a more powerful disinfectant than free chlorine and chlorine dioxide due to its highest oxidation potential of 2.07 V (Taylor et al., 2000; Cho et al., 2010). However, researchers found that double-layered gram-positives such as *Mycobacterium* survived and became predominant after ozonation (Lee and Deininger, 2000). In our study, gene copies and proportions of *Mycobacterium* and *Legionella* increased after ozonation (Figure 5 and Figure S3). Therefore, it is necessary to quantify potential pathogens using qPCR, especially in disinfected water. In addition, inactivation methods that are more effective should be used to guarantee drinking water safety.

### Temporal and spatial dynamics of microbial communities

**Figure 6:**
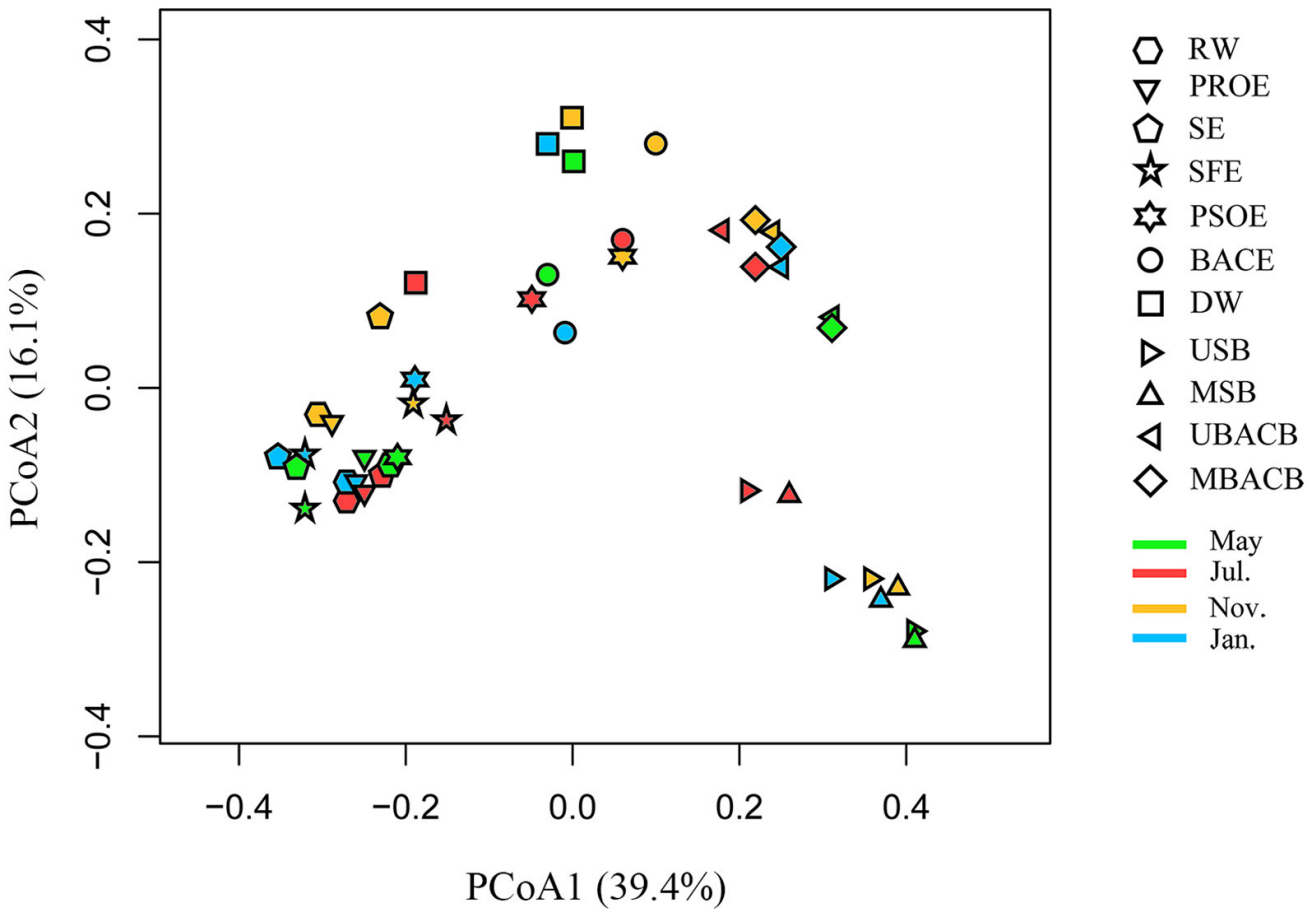
Principal coordinates analysis (PCoA) of the samples using weighted UniFrac metrics. The abbreviations of the samples are the same as used in Table 2.

The dynamics of community structures at all the sampling locations and time points are illustrated in Figure 6. Principal Axis 1 and Principal Axis 2 for PCoA represent 39.4 and 16.1% of the variation among the samples, respectively. Biofilm and water samples occupied divergent positions. The water samples clustered before post-ozonation, then distinctly separated during seasonal sampling after post-ozonation. Seasonal post-ozonation samples exhibited a dispersive distribution. The results from PERMANOVA revealed that the samples with no significant difference from two sampling sites included raw water and preozonation effluent, preozonation and sedimentation effluents, sedimentation and sand filtration effluents, sand filtration and post-ozonation effluents, post-ozonation and BAC effluents and BAC effluent and disinfected water (*P* > 0.05). All these samples with no significant difference had a feature of at least one sample from each sampling site clustering closely (Figure 6). According to PERMANOVA, disinfection was the only individual treatment step which significantly influenced bacterial community (*P* < 0.05) (Kim et al., 2013). Thus, the dynamics of the disinfected water samples were different from those of the other water samples (Figure 6). Alternatively, BAC biofilms separated from sand biofilms, but they each clustered despite different sampling depths and time. These biofilm dynamics revealed that biofilm bacterial communities were uniform from the surface to middle part of each filter, with high stability between different sampling times.

The bacterial community structure at each treatment step (except post-ozonation and BAC filtration effluents) was relatively stable during seasonal sampling. A reasonable explanation was that the community structure was governed by broader environmental conditions than temperature alone (Kim et al., 2013; Zwirglmaier et al., 2015). The dynamics of the microbial communities were mainly affected by the treatment processes rather than seasonal changes. A previous study also found that activated sludge communities were shaped by treatment processes (Lee et al., 2015). The susceptibility of the bacterial community in post-ozonation effluent to seasonal changes may be due to the great impacts of temperature on the solubility and decay of ozone (Gardoni et al., 2012). Compared with the water quality changes from the high ozone dosage in post-ozonation (1 mg/L), preozonation (with the lower dosage of 0.5 mg/L) changed water quality little, leading to a bacterial community in preozonation effluent that is more stable with regard to seasonal changes. The clustering of samples before post-ozonation illustrated the negligible effects of preozonation, flocculation, sedimentation and sand filtration on shifting the microbiome (Li et al., 2017; Xu et al., 2017). The samples before BAC filtration separated from the samples after BAC filtration, which was consistent with the previous find that filtration shaped the bacterial community in the corresponding effluent (Lautenschlager et al., 2014).

### Variations of pollutant degradation functions during the treatment processes

**Figure 7:**
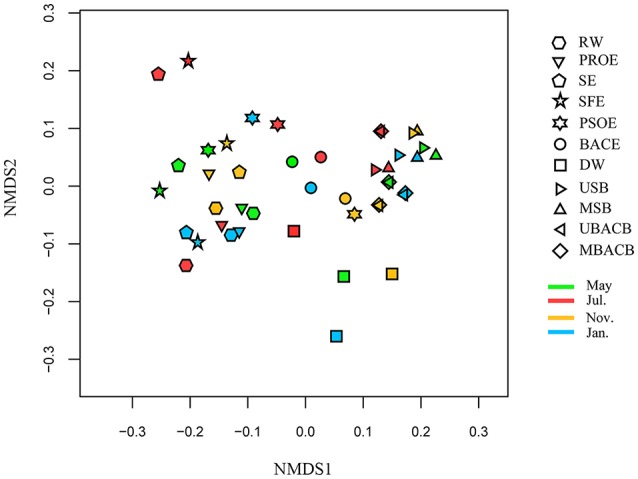
NMDS analysis based on predictive functional genes. The abbreviations of the samples are the same as used in Table 2.

The results of the NMDS analysis of the samples based on predictive functional genes is illustrated in Figure 7. The water samples dispersed without obvious clustering. These functional profiles displayed no seasonal associations. The functions of biofilm samples were more stable than those of water samples In addition, the clustering of sand and BAC biofilms implied their similarity in function despite their discrepancy in community composition, which may be attributed to functional redundancy within communities (Allison and Martiny, 2008).

**Figure 8:**
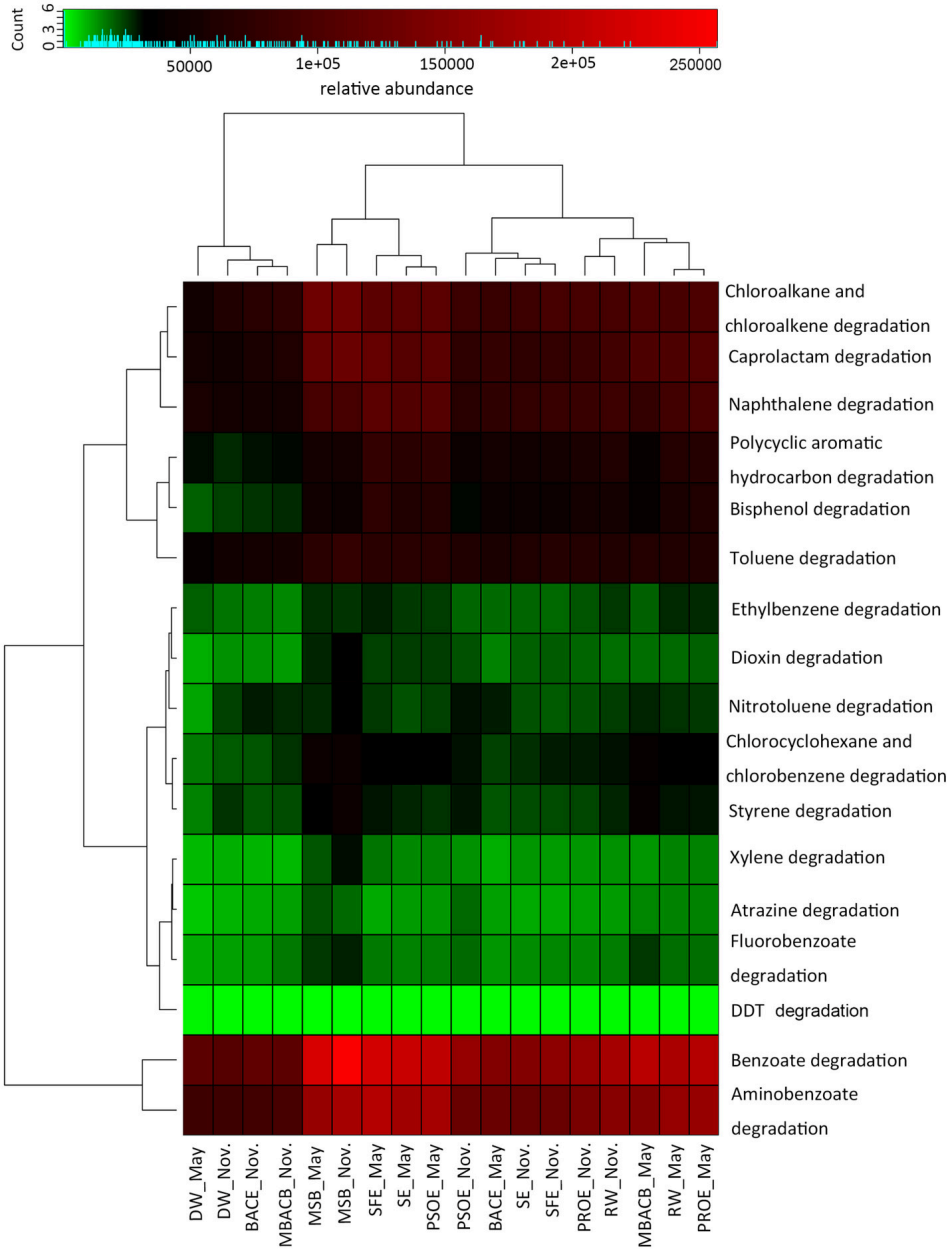
Heatmap of functional genes relating to pollutant degradation predicted using PICRUSt along the treatment processes in November and May. The relative abundance of each functional gene is indicated by color intensity with the legend at the top. The upper and left panels show the hierarchical clustering. The abbreviations of the samples are the same as used in Table 2. DDT is short for 2,2-bis(4-chlorophenyl)-1,1,1-trichloroethane.

The KEGG pathway-related functional profiles that were predicted using PICRUSt can be classified into several functional groups, including metabolism, genetic information processing, cellular processes and environmental information processing. Some functions relating to pollutant degradation, such as atrazine degradation, bisphenol degradation and naphthalene degradation, were identified in the xenobiotics degradation and metabolism profiles of the samples. Variations of pollutant degradation functions along the treatment processes in November and May are illustrated in Figure 8. The numbers of sequences assigned to pollutant degradation were the lowest and highest in November and May, respectively. The ability to degrade each type of pollutants differed in abundance. High percentages of sequences were assigned to aminobenzoate, benzoate, caprolactam, chloroalkane, and naphthalene degradation, while low percentage of sequences were assigned to 2,2-bis(4-chlorophenyl)-1,1,1-trichloroethane (DDT) degradation. The abundance of genes involved in pollutant biodegradation generally showed decreased during the treatment processes. These functional profiles, except for DDT degradation, were most abundance on the sand filter. These findings revealed that microbes in the treatment processes were possibly involved in the degradation of a variety of organic pollutants and that the role of sand filtration in pollutant degradation may be underestimated. A previous study found that toxic chemicals increased abundance of microbial metabolic enzymes and pathways (Lu et al., 2017). The correlation between pollutant degradation functions and the concentration of the corresponding pollutants needs further research.

### Relationships between bacterial communities and water quality parameters

**Figure 9:**
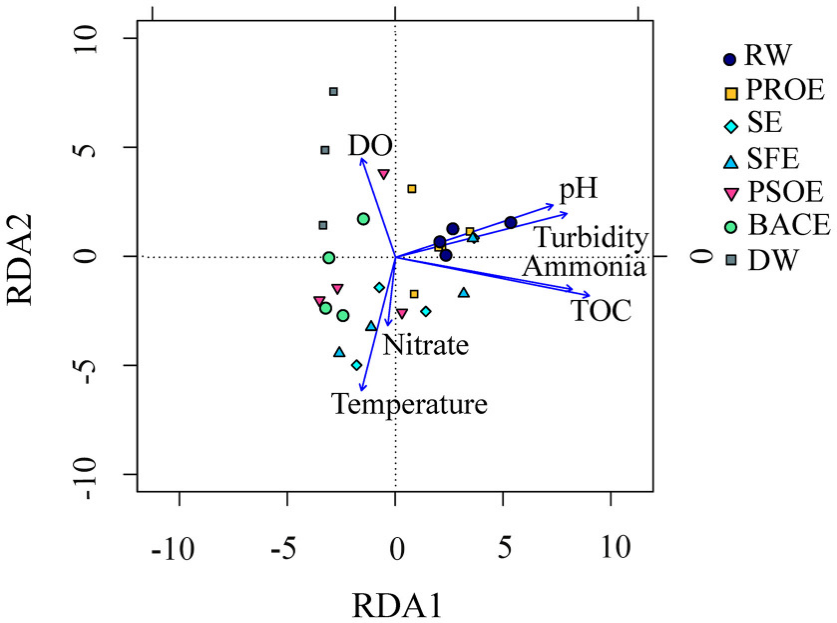
Redundancy analysis (RDA) of main bacterial phyla and environmental parameters. The abbreviations of the samples are the same as used in Table 2.

RDA was used to analyze the relationships between environmental parameters and bacterial community structures (Figure 9). RDA showed that turbidity, ammonia nitrogen and TOC exerted significant effects on community profiles (*p* < 0.01). Ammonia nitrogen and TOC were related to nutrition conditions (Zhang et al., 2009; Liao et al., 2013). Turbidity adjusted the proportions of particle–associated and free-living microbial communities (Dang and Lovell, 2016). The bacterial community structures in raw water and preozonation effluent samples positively correlated with pH, turbidity, ammonia nitrogen and TOC with seasonal changes. The correlation strength of preozonation effluent particularly varied during seasonal sampling. The bacterial community in disinfected water positively correlated with DO.

Table 3 shows that the relative abundances of bacterial phyla and proteobacterial classes were correlated to water quality parameters. Higher temperatures were more favorable for *Alphaproteobacteria, Nitrospirae* and *Gemmatimonadetes* (*p* < 0.05 or 0.01) but more detrimental for *Cyanobacteria* (*p* < 0.01). The relative abundances of *Actinobacteria, Cyanobacteria*, and *Planctomycetes* positively correlated with pH values (*p* < 0.05), whereas those of *Gammaproteobacteria, Deltaproteobacteria, Firmicutes, Acidobacteria, Nitrospirae, Gemmatimonadetes*, and *Euryarchaeota* negatively correlated (*p* < 0.05). *Actinobacteria, Planctomycetes* and *Verrucomicrobia* were powerful competitors in highly turbid water (*p* < 0.05), but *Alphaproteobacteria* was not (*p* < 0.05). *Actinobacteria, Planctomycetes*, and *Verrucomicrobia* positively correlated with ammonia nitrogen (*p* < 0.05). Conversely, *Firmicutes, Gammaproteobacteria, Deltaproteobacteria*, and *Euryarchaeota* negatively correlated with ammonia nitrogen (*p* < 0.05). High DO levels facilitated *Cyanobacteria* growth (*p* < 0.01), but inhibited *Actinobacteria* and *Gemmatimonadetes*. *Actinobacteria* and *Verrucomicrobia* were present in higher relative abundances in high-TOC environments (*p* < 0.01), whereas *Firmicutes, Euryarchaeota, Gammaproteobacteria*, and *Deltaproteobacteria* were more competitive in low-TOC environments (*p* < 0.05).

**Table 3:** Correlations between the water quality parameters and the relative abundance of predominant bacterial phyla and proteobacterial classes.

**Classification**	**Temperature**	**pH**	**Turbidity**	**Ammonia nitrogen**	**DO**	**TOC**
*Proteobacteria*	0.30	−0.51^b^	−0.57^b^	−0.44^a^	−0.06	−0.48^a^
*Alphaproteobacteria*	0.40^a^	−0.26	−0.38^a^	−0.18	−0.22	−0.23
*Gammaproteobacteria*	−0.05	−0.49^b^	−0.24	−0.47^a^	0.13	−0.41^a^
*Deltaproteobacteria*	0.28	−0.52^b^	−0.35	−0.42^a^	−0.16	−0.58^b^
*Actinobacteria*	−0.01	0.44^a^	0.54^b^	0.59^b^	−0.22^b^	0.68^c^
*Cyanobacteria*	−0.59^b^	0.72^c^	0.06	0.22	0.55^b^	0.06
*Firmicutes*	−0.09	−0.48^a^	−0.32	−0.58^b^	0.11	−0.52^b^
*Planctomycetes*	−0.14	0.49^b^	0.51^b^	0.47^a^	0.18	0.32
*Acidobacteria*	0.26	−0.45^a^	−0.37	−0.31	0.00	−0.31
*Verrucomicrobia*	0.16	0.35	0.93^d^	0.70^c^	−0.25	0.69^c^
*Nitrospirae*	0.44^a^	−0.40^a^	−0.25	−0.19	−0.18	−0.28
*Gemmatimonadetes*	0.59^b^	−0.49^b^	−0.21	−0.28	−0.40^a^	−0.16
*Euryarchaeota*	−0.03	−0.50^b^	−0.28	−0.53^b^	0.06	−0.48^a^

a Significance < 0.05,

b Significance < 0.01,

c Significance < 0.0001,

d *Significance < 1E-10*.

*Alphaproteobacteria* were relatively more abundant at high temperatures and low turbidity. Higher TOC and turbidity in raw water favored *Actinobacteria* (Glöckner et al., 2000; Wu et al., 2007), resulting in a larger number of *Actinobacteria* than *Alphaproteobacteria*. The decrease in TOC and turbidity during treatment decreased the *Actinobacteria* population. Similarly, *Verrucomicrobia* was relatively abundant in raw water and BAC filter, where the TOC concentrations were higher. By contrast, *Firmicutes, Euryarchaeota, Gammaproteobacteria*, and *Deltaproteobacteria* adapted to low-TOC environments, becoming highly abundant in treated water. In addition, low temperatures promoted the growth of *Cyanobacteria*, which is inconsistent with the previous reports of lake warming stimulating the growth of *Cyanobacteria* (Thomas and Litchman, 2016). The discrepancy may result from variations in the optimal growth temperatures of different *Cyanobacteria* (Lurling et al., 2013).

## Conclusions

The drinking water treatment processes harbored a high diversity of bacteria. *Proteobacteria, Actinobacteria, Acidobacteria, Planctomycetes*, and *Firmicutes* were dominant bacterial phyla. Disinfection significantly influenced bacterial community structures, while other treatment processes synergized with their sequential processes to influence communities. The bacterial community composition in post-ozonation effluent, BAC effluent and disinfected water varied with seasonal changes. Bacterial communities in water and biofilms differed, and the latter mainly depended on filter materials. In contrast, biofilms on different filters have similar functional composition and similarly high stability. Although the water quality parameters of source water varied greatly in different seasons, the quality of the treated water remained relatively stable. Sand and BAC filtration effectively removed dissolved organic matters. PICRUSt analysis showed that functional genes related to degradation of some pollutants were widely distributed throughout the treatment processes, especially on sand and BAC filters. The two genera, *Mycobacterium* and *Legionella*, were quantified in the treatment processes. Chlorine was effective in removing most bacteria including some potential pathogens with the exception of *Mycobacterium*. Bacterial composition was determined by the interaction of all the water quality parameters, among which turbidity, ammonia nitrogen and TOC were the most important factors. This study was a comprehensive investigation into variations in microbial communities in a full-scale drinking water treatment plant during four representative months. Overall, bacteria in the treatment processes constituted a relatively stable bacterial community structure that contributed to water purification. However, potential pathogens, especially those resistant to disinfectants, posed a threat to public's health.

## Author contributions

SY and LL conceived and supervised the study. QL and GL designed the experiments. QL, ZG, and YY performed the experiments. QL, ZL, and GL analyzed the data. QL wrote the paper. LR, QX, and ML revised the paper.

### Conflict of interest statement

The authors declare that the research was conducted in the absence of any commercial or financial relationships that could be construed as a potential conflict of interest.

## Abbreviations

BAC: Biological Activated Carbon
qPCR: Quantitative Polymerase Chain Reaction
RDA: Redundancy Analysis
TOC: Total Organic Carbon
O_3_-BAC: Ozone-Biological Activated Carbon
DO: Dissolved Oxygen
NGS: Next Generation Sequencing
PICRUSt: Phylogenetic Investigation of Communities by Reconstruction of Unobserved States
OTUs: Operational Taxonomic Units
ACE: Abundance-based Coverage Estimator
PCoA: Principal Coordinate Analysis
ANOVA: Analysis of Variance
KEGG: Kyoto Encyclopedia of Genes and Genomes
NSTI: Nearest Sequenced Taxon Index
NMDS: Non-metric Multidimensional Scaling
PERMANOVA: Permutational Multivariate Analysis of Variance.

## References

[B1] Allison S. D. Martiny J. B. (2008). Resistance, resilience, and redundancy in microbial communities. Proc. Natl. Acad. Sci. U.S.A. 105(Suppl. 1), 11512–11519. 10.1073/pnas.0801925105 18695234 PMC2556421

[B2] Anderson M. J. Walsh D. C. I. (2013). PERMANOVA, ANOSIM, and the Mantel test in the face of heterogeneous dispersions: what null hypothesis are you testing? Ecol. Monogr. 83, 557–574. 10.1890/12-2010.1

[B3] APHA (2011). Standard Methods for the Examination of Water and Wastewater. Washington, DC: American Public Health Association.

[B4] Belila A. El-Chakhtoura J. Otaibi N. Muyzer G. Gonzalez-Gil G. Saikaly P. E.. (2016). Bacterial community structure and variation in a full-scale seawater desalination plant for drinking water production. Water Res. 94, 62–72. 10.1016/j.watres.2016.02.039 26925544

[B5] Berjeaud J. M. Chevalier S. Schlusselhuber M. Portier E. Loiseau C. Aucher W.. (2016). *Legionella pneumophila*: the paradox of a highly sensitive opportunistic waterborne pathogen able to persist in the environment. Front. Microbiol. 7:486. 10.3389/fmicb.2016.00486. 27092135 PMC4824771

[B6] Brinkman N. E. Haugland R. A. Wymer L. J. Byappanahalli M. Whitman R. L. Vesper S. J. (2003). Evaluation of a rapid, quantitative real-time PCR method for enumeration of pathogenic Candida cells in water. Appl. Environ. Microbiol. 69, 1775–1782. 10.1128/AEM.69.3.1775-1782.2003 12620869 PMC150045

[B7] Camper A. K. LeChevallier M. Broadaway S. McFeters G. (1986). Bacteria associated with granular activated carbon particles in drinking water. Appl. Environ. Microbiol. 52, 434–438. 3767356 10.1128/aem.52.3.434-438.1986 PMC203552

[B8] Caporaso J. G. Kuczynski J. Stombaugh J. Bittinger K. Bushman F. D. Costello E. K.. (2010). QIIME allows analysis of high-throughput community sequencing data. Nat. Methods 7, 335–336. 10.1038/nmeth.f.303 20383131 PMC3156573

[B9] Chen C. Khaleel S. S. Huang H. Wu C. H. (2014). Software for pre-processing Illumina next-generation sequencing short read sequences. Source Code Biol. Med. 9:8. 10.1186/1751-0473-9-8 24955109 PMC4064128

[B10] Cho M. Kim J. Kim J. Y. Yoon J. Kim J. H. (2010). Mechanisms of Escherichia coli inactivation by several disinfectants. Water Res. 44, 3410–3418. 10.1016/j.watres.2010.03.017 20427068

[B11] Cole J. R. Wang Q. Cardenas E. Fish J. Chai B. Farris R. J.. (2009). The Ribosomal Database Project: improved alignments and new tools for rRNA analysis. Nucleic Acids Res. 37, D141–D145. 10.1093/nar/gkn879 19004872 PMC2686447

[B12] Dang H. Y. Lovell C. R. (2016). Microbial surface colonization and biofilm development in marine environments. Microbiol. Mol. Biol. Rev. 80, 91–138. 10.1128/mmbr.00037-15 26700108 PMC4711185

[B13] Edgar R. C. (2010). Search and clustering orders of magnitude faster than BLAST. Bioinformatics 26, 2460–2461. 10.1093/bioinformatics/btq461 20709691

[B14] Edgar R. C. Haas B. J. Clemente J. C. Quince C. Knight R. (2011). UCHIME improves sensitivity and speed of chimera detection. Bioinformatics 27, 2194–2200. 10.1093/bioinformatics/btr381 21700674 PMC3150044

[B15] Eiler A. Langenheder S. Bertilsson S. Tranvik L. J. (2003). Heterotrophic bacterial growth efficiency and community structure at different natural organic carbon concentrations. Appl. Environ. Microbiol. 69, 3701–3709. 10.1128/aem.69.7.3701-3709.2003 12839735 PMC165184

[B16] Feng L. Liu S. Wu W. Ma J. Li P. Xu H.. (2016). Dominant genera of cyanobacteria in Lake Taihu and their relationships with environmental factors. J. Microbiol. 54, 468–476. 10.1007/s12275-016-6037-4 27350612

[B17] Fonseca A. C. Summers R. S. Hernandez M. T. (2001). Comparative measurements of microbial activity in drinking water biofilters. Water Res. 35, 3817–3824. 10.1016/s0043-1354(01)00104-x 12230164

[B18] Gardoni D. Vailati A. Canziani R. (2012). Decay of ozone in water: a review. Ozone Sci. Eng. 34, 233–242. 10.1080/01919512.2012.686354

[B19] Glöckner F. O. Zaichikov E. Belkova N. Denissova L. Pernthaler J. Pernthaler A.. (2000). Comparative 16S rRNA analysis of lake bacterioplankton reveals globally distributed phylogenetic clusters including an abundant group of actinobacteria. Appl. Environ. Microbiol. 66, 5053–5065. 10.1128/aem.66.11.5053-5065.2000 11055963 PMC92419

[B20] Gomez-Alvarez V. Revetta R. P. Domingo J. W. S. (2012). Metagenomic analyses of drinking water receiving different disinfection treatments. Appl. Environ. Microbiol. 78, 6095–6102. 10.1128/aem.01018-12 22729545 PMC3416622

[B21] Hayes-Larson E. L. Mitch W. A. (2010). Influence of the method of reagent addition on dichloroacetonitrile formation during chloramination. Environ. Sci. Technol. 44, 700–706. 10.1021/es9025112 20000677

[B22] Hedegaard M. J. Albrechtsen H. J. (2014). Microbial pesticide removal in rapid sand filters for drinking water treatment–potential and kinetics. Water Res. 48, 71–81. 10.1016/j.watres.2013.09.024 24112625

[B23] Hirai J. Nagai S. Hidaka K. (2017). Evaluation of metagenetic community analysis of planktonic copepods using Illumina MiSeq: comparisons with morphological classification and metagenetic analysis using Roche 454. PLoS ONE 12:e0181452. 10.1371/journal.pone.0181452 28715458 PMC5513544

[B24] Holinger E. P. Ross K. A. Robertson C. E. Stevens M. J. Harris J. K. Pace N. R. (2014). Molecular analysis of point-of-use municipal drinking water microbiology. Water Res. 49, 225–235. 10.1016/j.watres.2013.11.027 24333849

[B25] Hunt N. K. Marinas B. J. (1999). Inactivation of Escherichia coli with ozone: chemical and inactivation kinetics. Water Res. 33, 2633–2641. 10.1016/s0043-1354(99)00115-3

[B26] Hutalle-Schmelzer K. M. Zwirnmann E. Krüeger A. Grossart H. P. (2010). Enrichment and cultivation of pelagic bacteria from a humic lake using phenol and humic matter additions. FEMS Microbiol. Ecol. 72, 58–73. 10.1111/j.1574-6941.2009.00831.x 20459514

[B27] Kim T. G. Yun J. Hong S. H. Cho K. S. (2014). Effects of water temperature and backwashing on bacterial population and community in a biological activated carbon process at a water treatment plant. Appl. Microbiol. Biotechnol. 98, 1417–1427. 10.1007/s00253-013-5057-9 23836347

[B28] Kim T. S. Jeong J. Y. Wells G. F. Park H. D. (2013). General and rare bacterial taxa demonstrating different temporal dynamic patterns in an activated sludge bioreactor. Appl. Microbiol. Biotechnol. 97, 1755–1765. 10.1007/s00253-012-4002-7 22526777

[B29] Langille M. G. Zaneveld J. Caporaso J. G. McDonald D. Knights D. Reyes J. A.. (2013). Predictive functional profiling of microbial communities using 16S rRNA marker gene sequences. Nat. Biotechnol. 31, 814–821. 10.1038/nbt.2676 23975157 PMC3819121

[B30] LaPara T. M. Wilkinson K. Strait J. M. Hozalski R. M. Sadowksy M. J. Hamilton M. J. (2015). The bacterial communities of full-scale biologically active, granular activated carbon filters are stable and diverse and potentially contain novel ammonia-oxidizing microorganisms. Appl. Environ. Microbiol. 81, 6864–6872. 10.1128/aem.01692-15 26209671 PMC4561712

[B31] Lautenschlager K. Hwang C. Ling F. Liu W. T. Boon N. Köster O.. (2014). Abundance and composition of indigenous bacterial communities in a multi-step biofiltration-based drinking water treatment plant. Water Res. 62, 40–52. 10.1016/j.watres.2014.05.035 24937356

[B32] Lautenschlager K. Hwang C. Liu W. T. Boon N. Köster O. Vrouwenvelder H.. (2013). A microbiology-based multi-parametric approach towards assessing biological stability in drinking water distribution networks. Water Res. 47, 3015–3025. 10.1016/j.watres.2013.03.002 23557697

[B33] Lee J. Deininger R. A. (2000). Survival of bacteria after ozonation. Ozone Sci. Eng. 22, 65–75. 10.1080/01919510008547229

[B34] Lee S. H. Kang H. J. Park H. D. (2015). Influence of influent wastewater communities on temporal variation of activated sludge communities. Water Res. 73, 132–144. 10.1016/j.watres.2015.01.014 25655320

[B35] LeLacheur R. M. Glaze W. H. (1996). Reactions of ozone and hydroxyl radicals with serine. Environ. Sci. Technol. 30, 1072–1080. 10.1021/es940544z

[B36] Li C. Ling F. Zhang M. Liu W. T. Li Y. Liu W. (2017). Characterization of bacterial community dynamics in a full-scale drinking water treatment plant. J. Environ. Sci. 51, 21–30. 10.1016/j.jes.2016.05.042 28115132

[B37] Li X. Upadhyaya G. Yuen W. Brown J. Morgenroth E. Raskin L. (2010). Changes in the structure and function of microbial communities in drinking water treatment bioreactors upon addition of phosphorus. Appl. Environ. Microbiol. 76, 7473–7481. 10.1128/aem.01232-10. 20889793 PMC2976211

[B38] Li Y. P. Tang C. Y. Yu Z. B. Acharya K. (2014). Correlations between algae and water quality: factors driving eutrophication in Lake Taihu, China. Int. J. Environ. Sci. Technol. 11, 169–182. 10.1007/s13762-013-0436-4.

[B39] Liao X. Chen C. Wang Z. Wan R. Chang C.-H. Zhang X. (2013). Pyrosequencing analysis of bacterial communities in drinking water biofilters receiving influents of different types. Process Biochem. 48, 703–707. 10.1016/j.procbio.2013.02.033

[B40] Lin W. Yu Z. Zhang H. Thompson I. P. (2014). Diversity and dynamics of microbial communities at each step of treatment plant for potable water generation. Water Res. 52, 218–230. 10.1016/j.watres.2013.10.071 24268295

[B41] Lindstrom E. S. (2000). Bacterioplankton community composition in five lakes differing in trophic status and humic content. Microb. Ecol. 40, 104–113. 10.1007/s002480000036 11029079

[B42] Loman N. J. Misra R. V. Dallman T. J. Constantinidou C. Gharbia S. E. Wain J.. (2012). Performance comparison of benchtop high-throughput sequencing platforms. Nat. Biotechnol. 30, 434–439. 10.1038/nbt.2198 22522955

[B43] Lu X. M. Chen C. Zheng T. L. (2017). Metagenomic insights into effects of chemical pollutants on microbial community composition and function in estuarine sediments receiving polluted river water. Microb. Ecol. 73, 791–800. 10.1007/s00248-016-0868-8 27744476

[B44] Lurling M. Eshetu F. Faassen E. J. Kosten S. Huszar V. L. M. (2013). Comparison of cyanobacterial and green algal growth rates at different temperatures. Freshw. Biol. 58, 552–559. 10.1111/j.1365-2427.2012.02866.x

[B45] Martiny A. C. Albrechtsen H. J. Arvin E. Molin S. (2005). Identification of bacteria in biofilm and bulk water samples from a nonchlorinated model drinking water distribution system: detection of a large nitrite-oxidizing population associated with Nitrospira spp. Appl. Environ. Microbiol. 71, 8611–8617. 10.1128/aem.71.12.8611-8617.2005 16332854 PMC1317318

[B46] Mi Z. Dai Y. Xie S. Chen C. Zhang X. (2015). Impact of disinfection on drinking water biofilm bacterial community. J. Environ. Sci. 37, 200–205. 10.1016/j.jes.2015.04.008 26574105

[B47] Mir J. Morató J. Ribas F. (1997). Resistance to chlorine of freshwater bacterial strains. J. Appl. Microbiol. 82, 7–18. 10.1111/j.1365-2672.1997.tb03292.x 9113873

[B48] Nazarian E. J. Bopp D. J. Saylors A. Limberger R. J. Musser K. A. (2008). Design and implementation of a protocol for the detection of Legionella in clinical and environmental samples. Diagn. Microbiol. Infect. Dis. 62, 125–132. 10.1016/j.diagmicrobio.2008.05.004 18621500

[B49] Newton R. J. Jones S. E. Eiler A. McMahon K. D. Bertilsson S. (2011). A guide to the natural history of freshwater lake bacteria. Microbiol. Mol. Biol. Rev. 75, 14–49. 10.1128/mmbr.00028-10 21372319 PMC3063352

[B50] Noyce G. L. Fulthorpe R. Gorgolewski A. Hazlett P. Honghi T. Basiliko N. (2016). Soil microbial responses to wood ash addition and forest fire in managed Ontario forests. Appl. Soil Ecol. 107, 368–380. 10.1016/j.apsoil.2016.07.006

[B51] Pang Y. C. Xi J. Y. Xu Y. Huo Z. Y. Hu H. Y. (2016). Shifts of live bacterial community in secondary effluent by chlorine disinfection revealed by Miseq high-throughput sequencing combined with propidium monoazide treatment. Appl. Microbiol. Biotechnol. 100, 6435–6446. 10.1007/s00253-016-7452-5 27005415

[B52] Parfenova V. V. Gladkikh A. S. Belykh O. I. (2013). Comparative analysis of biodiversity in the planktonic and biofilm bacterial communities in Lake Baikal. Microbiology 82, 91–101. 10.1134/s0026261713010128

[B53] Pinto A. J. Xi C. Raskin L. (2012). Bacterial community structure in the drinking water microbiome is governed by filtration processes. Environ. Sci. Technol. 46, 8851–8859. 10.1021/es302042t 22793041

[B54] Prest E. I. Hammes F. van Loosdrecht M. C. Vrouwenvelder J. S. (2016). Biological stability of drinking water: controlling factors, methods, and challenges. Front. Microbiol. 7:45. 10.3389/fmicb.2016.00045 26870010 PMC4740787

[B55] Proctor C. R. Hammes F. (2015). Drinking water microbiology - from measurement to management. Curr. Opin. Biotechnol. 33, 87–94. 10.1016/j.copbio.2014.12.014 25578740

[B56] Qin Y. Y. Li D. T. Yang H. (2007). Investigation of total bacterial and ammonia-oxidizing bacterial community composition in a full-scale aerated submerged biofilm reactor for drinking water pretreatment in China. FEMS Microbiol. Lett. 268, 126–134. 10.1111/j.1574-6968.2006.00571.x 17263855

[B57] Radomski N. Lucas F. S. Moilleron R. Cambau E. Haenn S. Moulin L. (2010). Development of a real-time qPCR method for detection and enumeration of *Mycobacterium* spp. in surface water. Appl. Environ. Microbiol. 76, 7348–7351. 10.1128/AEM.00942-10 20851986 PMC2976254

[B58] Schloss P. D. Westcott S. L. Ryabin T. Hall J. R. Hartmann M. Hollister E. B.. (2009). Introducing mothur: open-source, platform-independent, community-supported software for describing and comparing microbial communities. Appl. Environ. Microbiol. 75, 7537–7541. 10.1128/aem.01541-09 19801464 PMC2786419

[B59] Schmeisser C. Stöckigt C. Raasch C. Wingender J. Timmis K. N. Wenderoth D. F.. (2003). Metagenome survey of biofilms in drinking-water networks. Appl. Environ. Microbiol. 69, 7298–7309. 10.1128/aem.69.12.7298-7309.2003 14660379 PMC309957

[B60] Schmieder R. Edwards R. (2011). Quality control and preprocessing of metagenomic datasets. Bioinformatics 27, 863–864. 10.1093/bioinformatics/btr026 21278185 PMC3051327

[B61] Servais P. Billen G. Bouillot P. (1994). Biological colonization of granular activated carbon filters in drinking-water treatment. J. Environ. Eng. 120, 888–899. 10.1061/(asce)0733-9372(1994)120:4(888)

[B62] Shu D. He Y. Yue H. Wang Q. (2016). Metagenomic and quantitative insights into microbial communities and functional genes of nitrogen and iron cycling in twelve wastewater treatment systems. Chem. Eng. J. 290, 21–30. 10.1016/j.cej.2016.01.024

[B63] Simoes L. C. Simoes M. (2013). Biofilms in drinking water: problems and solutions. RSC Adv. 3, 2520–2533. 10.1039/c2ra22243d

[B64] Sinclair L. Osman O. A. Bertilsson S. Eiler A. (2015). Microbial vommunity vomposition and diversity via 16S rRNA gene amplicons: evaluating the Illumina platform. PLoS ONE 10:116955. 10.1371/journal.pone.0116955 25647581 PMC4315398

[B65] Smith D. P. Peay K. G. (2014). Sequence depth, not PCR replication, improves ecological inference from next generation DNA sequencing. PLoS ONE 9:90234 10.1371/journal.pone.0090234 PMC3938664 24587293

[B66] Staley C. Gould T. J. Wang P. Phillips J. Cotner J. B. Sadowsky M. J. (2015). Species sorting and seasonal dynamics primarily shape bacterial communities in the Upper Mississippi River. Sci. Total Environ. 505, 435–445. 10.1016/j.scitotenv.2014.10.012 25461045

[B67] Stanish L. F. Hull N. M. Robertson C. E. Harris J. K. Stevens M. J. Spear J. R.. (2016). Factors influencing bacterial diversity and community composition in municipal drinking waters in the Ohio River Basin, USA. PLoS ONE 11:e157966. 10.1371/journal.pone.0157966 27362708 PMC4928833

[B68] Stewart M. H. Wolfe R. L. Means E. G. (1990). Assessment of the bacteriological activity associated with granular ativated carbon treatment of drinking water. Appl. Environ. Microbiol. 56, 3822–3829. 2082828 10.1128/aem.56.12.3822-3829.1990 PMC185074

[B69] Suzuki M. T. Taylor L. T. DeLong E. F. (2000). Quantitative analysis of small-subunit rRNA genes in mixed microbial populations via 5′-nuclease assays. Appl. Environ. Microbiol. 66, 4605–4614. 10.1128/aem.66.11.4605-4614.2000 11055900 PMC92356

[B70] Tan B. Ng C. Nshimyimana J. P. Loh L. L. Gin K. Y. H. Thompson J. R. (2015). Next-generation sequencing (NGS) for assessment of microbial water quality: current progress, challenges, and future opportunities. Front. Microbiol. 6:1027 10.3389/fmicb.2015.01027 26441948 PMC4585245

[B71] Taylor R. H. Falkinham J. O. Norton C. D. LeChevallier M. W. (2000). Chlorine, chloramine, chlorine dioxide, and ozone susceptibility of *Mycobacterium avium*. Appl. Environ. Microbiol. 66, 1702–1705. 10.1128/aem.66.4.1702-1705.2000 10742264 PMC92045

[B72] Thomas M. K. Litchman E. (2016). Effects of temperature and nitrogen availability on the growth of invasive and native cyanobacteria. Hydrobiologia 763, 357–369. 10.1007/s10750-015-2390-2

[B73] Unno T. (2015). Bioinformatic suggestions on MiSeq-based microbial community analysis. J. Microbiol. Biotechnol. 25, 765–770. 10.4014/jmb.1409.09057 25563415

[B74] Vaerewijck M. J. Huys G. Palomino J. C. Swings J. Portaels F. (2005). Mycobacteria in drinking water distribution systems: ecology and significance for human health. Fems Microbiol. Rev. 29, 911–934. 10.1016/j.femsre.2005.02.001 16219512

[B75] Wang H. Masters S. Edwards M. A. Falkinham J. O. III. Pruden A. (2014). Effect of disinfectant, water age, and pipe materials on bacterial and eukaryotic community structure in drinking water biofilm. Environ. Sci. Technol. 48, 1426–1435. 10.1021/es402636u 24401122

[B76] Wu X. Xi W. Y. Ye W. J. Yang H. (2007). Bacterial community composition of a shallow hypertrophic freshwater lake in China, revealed by 16S rRNA gene sequences. FEMS Microbiol. Ecol. 61, 85–96. 10.1111/j.1574-6941.2007.00326.x 17506827

[B77] Xu J. Tang W. Ma J. Wang H. (2017). Comparison of microbial community shifts in two parallel multi-step drinking water treatment processes. Appl. Microbiol. Biotechnol. 101, 5531–5541. 10.1007/s00253-017-8258-9 28396926

[B78] Yang B. M. Liu J. K. Chien C. C. Surampalli R. Y. Kao C. M. (2011). Variations in AOC and microbial diversity in an advanced water treatment plant. J. Hydrol. 409, 225–235. 10.1016/j.jhydrol.2011.08.022

[B79] Yang J. X. Ma J. Song D. Zhai X. D. Kong X. J. (2016). Impact of preozonation on the bioactivity and biodiversity of subsequent biofilters under low temperature conditions-A pilot study. Front. Env. Sci. Eng. 10:5 10.1007/s11783-016-0844-z

[B80] Yu S. Lin T. Chen W. (2014). Photocatalytic inactivation of particle-associated *Escherichia coli* using UV/TiO_2_. RSC Adv. 4, 31370–31377. 10.1039/C4RA04061A

[B81] Zeng D. N. Fan Z. Y. Chi L. Wang X. Qu W. D. Quan Z. X. (2013). Analysis of the bacterial communities associated with different drinking water treatment processes. World J. Microbiol. Biotechnol. 29, 1573–1584. 10.1007/s11274-013-1321-5 23515963

[B82] Zhang Y. Li P. Zhou L. (2015). Study on the release of HPC and particles in ozonation and biological activated carbon processes. Chem. Eng. J. 276, 37–43. 10.1016/j.cej.2015.04.062

[B83] Zhang Y. Love N. Edwards M. (2009). Nitrification in drinking water systems. Crit. Rev. Environ. Sci. Technol. 39, 153–208. 10.1080/10643380701631739

[B84] Zwart G. Crump B. C. Agterveld M. Hagen F. Han S. K. (2002). Typical freshwater bacteria: an analysis of available 16S rRNA gene sequences from plankton of lakes and rivers. Aquat. Microb. Ecol. 28, 141–155. 10.3354/ame028141

[B85] Zwirglmaier K. Keiz K. Engel M. Geist J. Raeder U. (2015). Seasonal and spatial patterns of microbial diversity along a trophic gradient in the interconnected lakes of the Osterseen Lake District, Bavaria. Front. Microbiol. 6:1168. 10.3389/fmicb.2015.01168 26579082 PMC4623418

