# Supplementary material for: Microbial Communities Shaped by Treatment Processes in a Drinking Water Treatment Plant and Their Contribution and Threat to Drinking Water Safety
Source: Front Microbiol. 2017 Dec 12;8:2465. doi: 10.3389/fmicb.2017.02465 (PMC5733044; doi:10.3389/fmicb.2017.02465)
Supplement: Supplementary file 1 [file Image1.PDF]

Supplementary Material

Microbial communities shaped by treatment processes in a drinking water treatment plant and their contribution and threat to drinking water safety

Qi Li, Shuili Yu*, Lei Li*, Guicai Liu, Zhengyang Gu, Minmin Liu, Zhiyuan Liu, Yubing Ye, Qing Xia, Liumo Ren

*** Correspondence:** Shuili Yu: ysl@tongji.edu.cn

Lei Li: lilei@tongji.edu.cn

1 Supplementary Figures

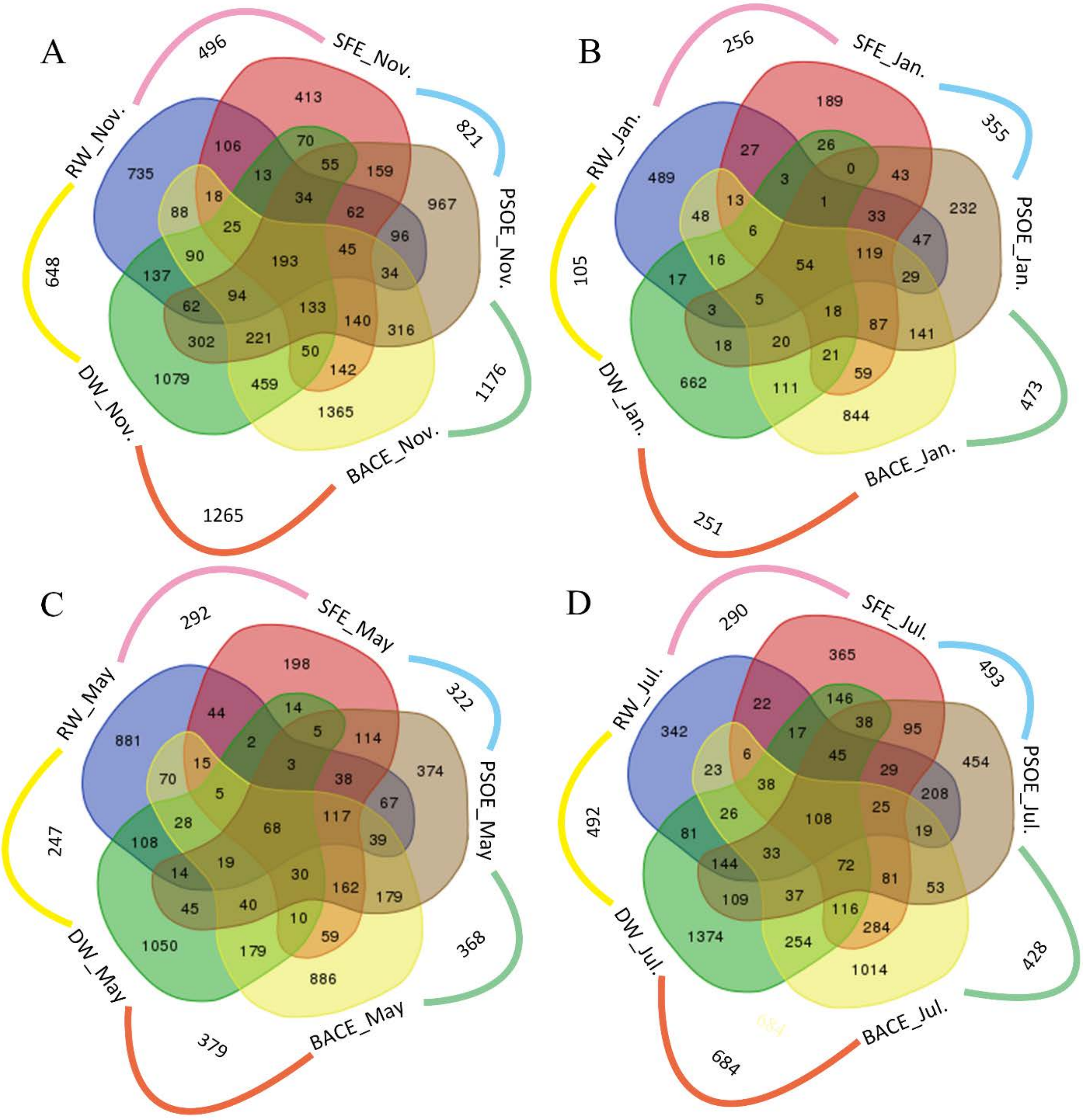

Figure S1. Venn diagrams showing the number of shared OTUs in the major steps of treatment processes in November **(A)**, January **(B)**, May **(C)** and July **(D)**.

.

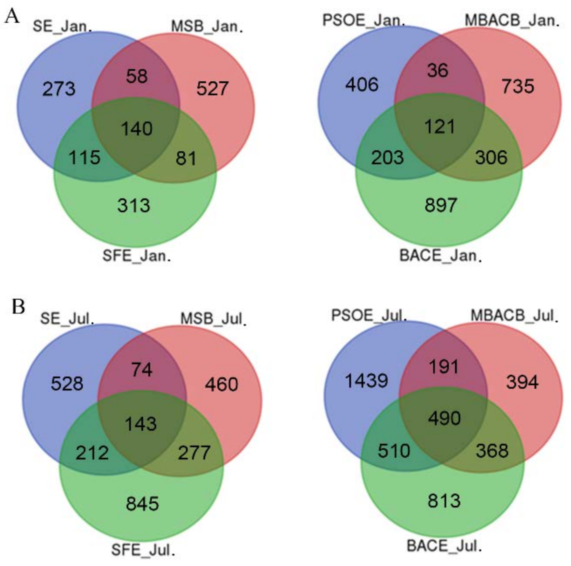

Figure S2. Venn diagrams showing the number of shared OTUs between filter biofilms and their corresponding influents and effluents in January **(A)** and July **(B)**

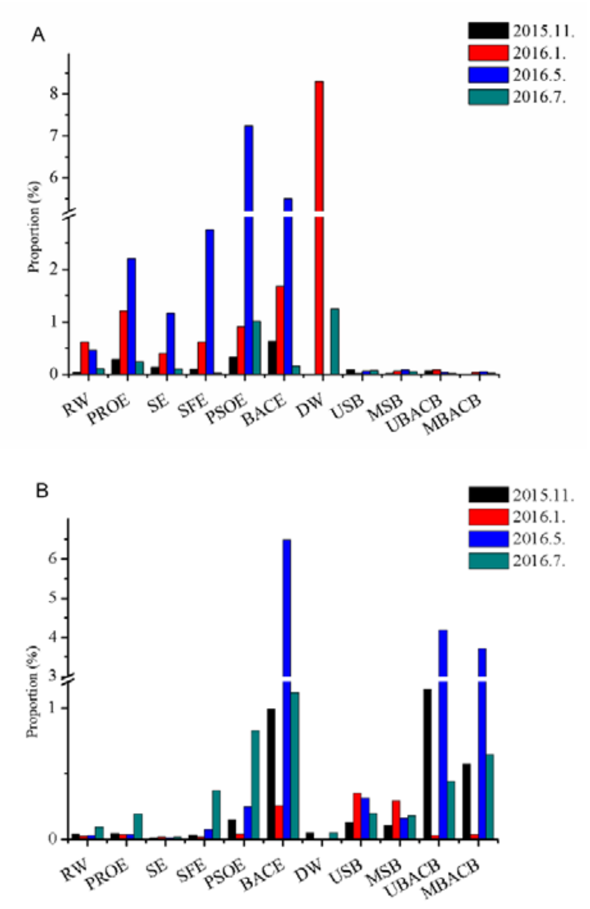

Figure S3. Ratios between the quantification of these genera (*Mycobacterium* spp. **(A)** and *Legionella* spp. **(B)**) and the total number of bacteria.
